# Increased serum lysyl oxidase-like 2 levels correlate with the degree of left atrial fibrosis in patients with atrial fibrillation

**DOI:** 10.1042/BSR20171332

**Published:** 2017-11-21

**Authors:** Yingming Zhao, Kangting Tang, Xu Tianbao, Junhong Wang, Jin Yang, Dianfu Li

**Affiliations:** 1 Department of Cardiology, The First Affiliated Hospital of Nanjing Medical University, Nanjing, China; 2 Department of Cardiology, The People’s Hospital of Kizilsu Kirghiz Autonomous Prefecture, Xinjiang, China; 3 Department of Medicine, Krannert Institute of Cardiology and Division of Cardiology, Indiana University School of Medicine, Indianapolis, Indiana, U.S.A.

**Keywords:** atrial fibrillation, atrial fibrosis, electroanatomic mapping, Lysyl oxidase-like 2 (LOXL2)

## Abstract

Atrial fibrillation (AF) progression is generally accompanied by increased atrial fibrosis and atrial structural remodeling. Lysyl oxidase-like 2 (LOXL2) is known to play an important role in many fibrotic conditions, including cardiac fibrosis. The present study aimed to explore the relationship between serum LOXL2 levels and AF. Fifty-four AF patients and 32 control subjects were enrolled in the study. High-density three-dimensional electroanatomic mapping was performed, and mean bipolar voltage was assessed in AF patients. LOXL2 levels were measured by enzyme-linked immunosorbent assay. All patients underwent echocardiography to assess left atrium size and left ventricle function. Serum LOXL2 levels were significantly elevated in AF patients compared with the control group (526.81 ± 316.82 vs 240.94 ± 92.51 pg/ml, *P*<0.01). In addition, serum LOXL2 level was significantly correlated with the size of the left atrium (LAD) (*r*^2^ = 0.38, *P*<0.01). Furthermore, the serum LOXL2 levels were significantly higher in AF patients with LAD ≥ 40 mm compared with those with LAD < 40 mm (664.34 ± 346.50 vs 354.90 ± 156.23 pg/ml, *P*<0.01). And the Spearman’s correlation analysis further revealed that the mean bipolar left atrial voltage was inversely correlated with the LOXL2 (*r*^2^ = −0.49, *P*<0.01) in AF patients. Multivariate regression analysis further demonstrated that serum LOXL2 [odds ratio (OR) 1.013, 95% confidence interval (CI) 1.002–1.024, *P*<0.05] and LAD (OR 1.704, 95% CI 1.131–2.568, *P*<0.01) were independent predictors of AF. In conclusion, serum LOXL2 levels were significantly elevated and were correlated with the degree of left atrial fibrosis in AF patients.

## Introduction

Atrial fibrillation (AF) is a common cardiac rhythm disturbance and is associated with increased morbidity and mortality [1]. AF occurs when structural and/or electrophysiologic abnormalities alter atrial tissue to promote abnormal impulse formation and/or propagation [2]. AF progression is generally accompanied by increased atrial fibrosis and atrial structural remodeling [3]. The association between AF and atrial fibrosis has been highlighted in several studies [4,5].

Lysyl oxidase-like-2 (LOXL2) belongs to the lysyl oxidase (LOX) family, which comprises Cu^2+^ and lysine tyrosylquinone (LTQ)-dependent amine oxidases. LOXL2 was proposed to function similarly to LOX in the extracellular matrix by promoting crosslinking of collagen and elastin [6,7]. One recent study focused on the association between LOXL2 levels and cardiac fibrosis in heart failure (HF) [8]. The present study showed that LOXL2 is up-regulated in the cardiac interstitium in diseased human hearts. LOXL2 levels were correlated with collagen crosslinking and cardiac dysfunction. Additionally, LOXL2 was elevated in the serum of HF patients. The study suggests a pathogenic role of LOXL2 in cardiac fibrosis and human HF.

However, LOXL2 as a potential biomarker for cardiac remodeling in an AF cohort has never been investigated. We hypothesized that higher levels of LOXL2, reflecting more extensive structural left atrium remodeling, could predict the degree of atrial fibrosis in AF patients. Thus, the present study aimed to explore the association between LOXL2 and AF.

## Methods

### Study population

Fifty-four paroxysmal or persistent AF consecutive patients with normal left ventricular function, who underwent a bipolar voltage map guided AF ablation between November, 2016 and April, 2017 were enrolled in the study. Thirty-two age- and gender-matched controls without AF who were hospitalized in our department were enrolled as the control patients. AF episodes either lasting >7 days or requiring termination by cardioversion, either with drugs or by direct-current cardioversion, were defined as persistent, whereas AF episodes self-terminating within 7 days were defined as paroxysmal. Exclusion criteria included noncardiac conditions with possible elevated LOXL2 levels such as liver cirrhosis, pulmonary fibrosis, autoimmune disease, and cancer. Patients with HF were also excluded from our study. Baseline demographic and clinical characteristics, including age, gender, complications, CHADS2 scores, and medication history, were recorded for all patients. N-terminal pro-B-type natriuretic peptide (NT-proBNP), serum creatinine (SCr), and alanine transaminase (ALT) were also recorded. Informed consent was obtained from each patient before enrollment. The study was in compliance with the principles outlined in the Declaration of Helsinki and approved by the Institutional Ethics Committee of Nanjing Medical University.

### Blood samples and LOXL2 measurements

After an overnight fast, blood samples were collected and immediately centrifuged and stored at −80°C until assayed. The frozen serum samples were rapidly thawed and brought to room temperature and assayed for the presence of human LOXL2 using enzyme-linked immunosorbent assay kits (MyBioscience, CA, U.S.A.), according to the manufacturer’s instructions. Serial dilutions of known concentrations of human LOXL2 were used to construct a standard curve of the analytes. The serum levels of LOXL2 from the samples were estimated by extrapolation from a log–log linear regression curve determined from the serially diluted human recombinant LOXL2.

### Echocardiography

All patients underwent transthoracic echocardiography to assess left atrium diameter and left ventricle (LV) function using standard views. The reported left atrial diameter (LAD) refers to left atrial anterior–posterior diameter, which was measured at end-systole on the M-mode image obtained from the parasternal long-axis view. The left ventricular function was assessed by the M-mode scanning of the left ventricular chamber, which was standardized by two-dimensional, short-axis views of the LV at the mid-papillary muscle level. The fractional shortening (FS) of the LV was defined as 100%, representing the relative change of left ventricular diameters during the cardiac cycle. The mean FS of the LV was determined by the average FS measurement of the left ventricular contraction over five beats.

### Electrophysiological study and voltage mapping

All AF patients underwent transesophageal echocardiography on the day of the study to exclude LA thrombus. Electrophysiological studies and ablations were carried out following the clinical routine in our center. In brief, a bipolar voltage map was carried out simultaneously with LA reconstruction, guided by the three-dimensional EnSite –NavX system (St. Jude Medical, St. Paul, MN, U.S.A.).

Intracardiac catheters were positioned as follows: (1) one decapolar coronary sinus (CS) catheter (2-5-2 mm interelectrode spacing, Daig Electrophysiology, Minnetonka, MN); (2) His-bundle/right-ventricular catheter (2-5-2 mm interelectrode spacing, Bard); and (3) circular mapping catheter: a deflectable decapolar circular catheter (1 mm ring electrode with 3 mm interelectrode spacing, A-Focus catheter, IBI, St. Jude Medical, Inc.) was advanced into the left atrium to create detailed LA and pulmonary vein (PV) geometries and acquire LA mapping points. The LA was accessed using two transseptal punctures.

Intracardiac electrograms and 12-lead surface ECG were recorded using a digital electrophysiological recording system (Prucka CardioLab, General Electric Health Care System Inc., Milwaukee, WI, U.S.A.) and intracardiac electrograms were filtered at 30–500 Hz. The LA electroanatomic map was created during sinus rhythm. The electrogram from the proximal CS was used as the reference signal during the mapping procedure. To minimize the points which do not have good contact, the interior and exterior projection distances were set at 5 mm to the surface of LA geometry. Voltage maps for each patient were constructed during sinus rhythm. Local voltage was defined as the amplitude from the peak-positive to the peak-negative deflection of the local bipolar electrogram. The left atrium was divided into six regions as previously described elsewhere [9]. The mean atrial voltage amplitudes among the entire left atrium were calculated.

### Statistical analysis

In the present study, continuous variables are shown as the mean ± standard deviation, and categorical data are presented as frequencies. Comparisons among the baseline characteristics and levels of LOXL2 in different groups were performed by independent Student’s *t*-tests, ANOVA, Fisher’s exact tests, or chi-square tests where appropriate. Univariate and multivariate binomial logistic regression analyses were performed to determine the independent predictors of AF. Spearman correlation analysis was performed to investigate factors related to the levels of LOXL2. A value of two-tailed *P*<0.05 was considered statistically significant. The statistical analysis was performed using SPSS software version 20.0 (SPSS Inc., Chicago, Illinois).

## Results

### Patient characteristics

Fifty-four AF patients with normal LV function and 34 age- and gender-matched control subjects were included in the study. Baseline demographic, clinical, laboratory, and echocardiographic parameters were compared between patients with AF and without AF and are presented in Table 1. Age, gender, and the prevalence of hypertension and diabetes mellitus were not significantly different between the two groups. Moreover, right atrium diameter (RAD), left ventricular end diastolic dimension (LVEDD), FS, left ventricular ejection fraction (LVEF), and the levels of SCr and ALT were also not significantly different. Compared with patients without AF, AF patients showed significantly higher left ventricular end systolic dimension (LVESD) (47.44 ± 4.55 vs 45.35 ± 4.51 mm, *P*<0.05), LAD (40.89 ± 6.37 vs 32.60 ± 3.46 mm, *P*<0.01), and NT-proBNP levels (290.36 ± 172.13 vs 194.90 ± 215.40 pg/ml, *P*<0.05).

**Table 1:** Baseline characteristics and comparison between the AF and control groups

Characteristic	AF group (*n*=54)	Control group (*n*=32)	*P* value
Age (years)	62.74 ± 15.94	57.63 ± 12.49	0.12
Male gender (%)	26(48.1%)	17(53.1%)	0.66
Hypertension (%)	30(55.6%)	16(50.0%)	0.62
Diabetes mellitus (%)	6(11.1%)	4(12.5%)	0.85
TIA or stroke (%)	12(22.2%)	2(6.25%)	0.05
CHADS2–VASC score			
0	12(22.2%)	N/A	N/A
1–2	27(50.0%)	N/A	N/A
≥3	15(27.8%)	N/A	N/A
Medication on admission			
Statin (%)	26(48.1%)	16(50.0%)	1.00
β-Blocker (%)	39(72.2%)	12(37.5%)	0.47
ACEI/ARB (%)	23(42.6%)	11(34.4%)	0.50
Warfarin (%)	34(63.0%)	0(0%)	N/A
NOAC (%)	11(20.4%)	0(0%)	N/A
Echocardiographic parameters			
LAD (mm)	40.89 ± 0.87	32.6 ± 0.63	0.00
LVEF (%)	65.60 ± 4.73	64.50 ± 3.24	0.26
LVEDD (mm)	47.44 ± 4.55	45.35 ± 4.51	0.05
LVESD (mm)	31.83 ± 4.65	29.93 ± 3.83	0.06
FS (%)	34.60 ± 2.59	34.36 ± 3.42	0.73
SCr (µmol/l)	75.37 ± 18.20	71.36 ± 14.29	0.30
ALT (U/l)	25.21 ± 21.43	25.09 ± 15.95	0.98
NT-proBNP (pg/ml)	290.36 ± 172.13	194.90 ± 215.40	0.00
LOXL2 (pg/ml)	526.81 ± 316.82	240.94 ± 92.51	0.00

### Serum LOXL2 levels and AF

**Figure 1:**
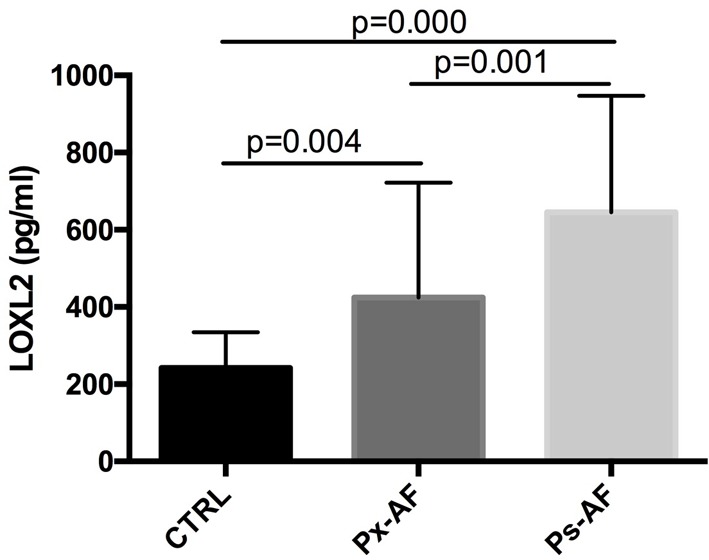
The serum LOXL2 in control and AF patients. Comparison of serum LOXL2 levels in the control group and patients with persistence AF (Ps-AF) and paroxysmal AF (Px-AF). The LOXL2 levels were significantly higher in Ps-AF and Px-AF patients than controls.

Serum LOXL2 levels (526.81 ± 316.82 pg/ml vs 240.94 ± 92.51 pg/ml, *P*<0.01) were significantly higher in patients with AF compared with the control group (Table 1). In addition, serum LOXL2 levels were increased in the patients with persistent AF (Ps-AF) than those patients with paroxysmal AF (Px-AF) (645.45 ± 301.37 pg/ml vs 434.54 ± 297.90 pg/ml, *P*<0.01) (Figure 1).

### Serum LOXL2 levels and LAD

**Figure 2:**
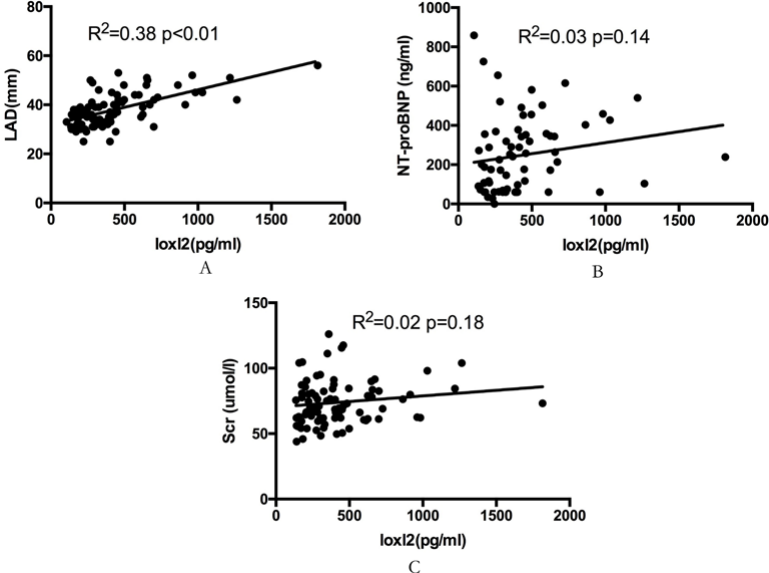
The correlation between LOXL2 and LAD, NT-proBNP and Scr in AF patients. The correlation between serum LOXL2 and LAD, NT-proBNP, and Scr. LOXL2 levels were significantly correlated with increased LAD level (*R*^2^ = 0.38, *P*<0.01) (**A**). The NT-proBNP (**B**), and Scr (**C**) levels were not associated with the LOXL2 levels.

**Figure 3:**
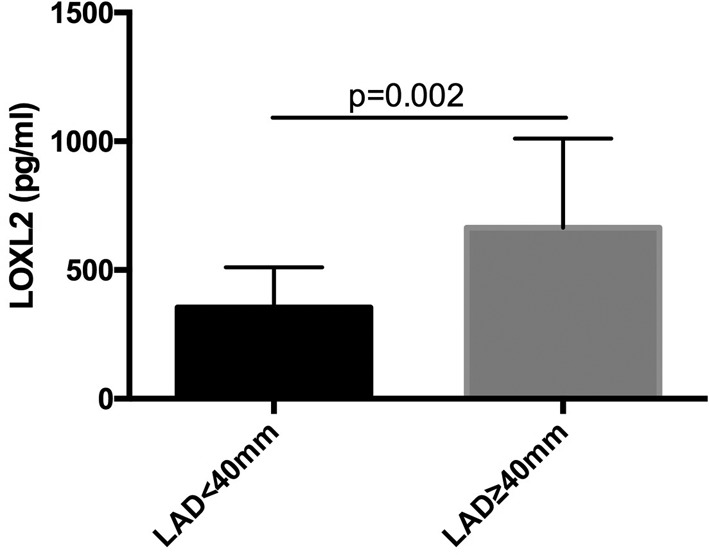
The level of serum LOXL2 in AF patients with LAD< 40mm and LAD>= 40mm. Comparison of LOXL2 levels in patients with LAD < 40 mm and patients with LAD ≥ 40 mm. The LOXL2 levels were significantly higher in AF patients with larger LAD.

In Spearman’s correlation analysis, LAD was significantly correlated with serum LOXL2 levels (*r*^2^ = 0.38, *P*<0.01) (Figure 2) in patients with AF. Meanwhile, no correlation was found between LOXL2 and the level of NT-proBNP or SCr (Figure 2). Furthermore, the serum LOXL2 levels were also significantly higher in AF patients with LAD ≥ 40 mm compared with the patients with LAD < 40 mm (664.34 ± 346.50 pg/ml vs 354.90 ± 156.23 pg/ml, *P*<0.01, Figure 3).

### Serum LOXL2 levels and the mean bipolar voltage in left atrium

**Figure 4:**
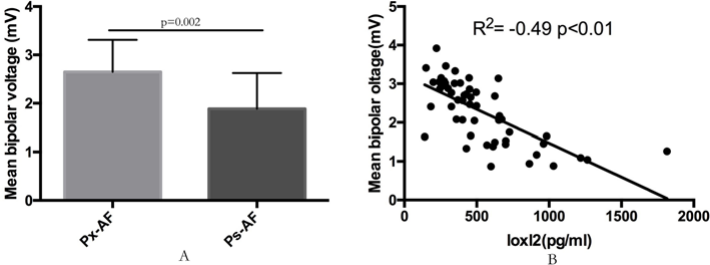
The correlation between serum LOXL2 and left atrial mean bipolar voltage in AF patients. The correlation between serum LOXL2 and mean bipolar voltage in the left atrial of Ps-AF and Px-AF patients. The mean bipolar voltage of the left atrium was significantly decreased in Ps-AF patients than those in Ps-AF patients (**A**). LOXL2 levels were significantly correlated with the mean bipolar voltage in AF patients (*R*^2^ = −0.49, *P*<0.01) (**B**).

The overall mean bipolar voltage of LA was significantly reduced in persistent AF when compared with the paroxysmal AF patients (1.89 ± 0.74 vs 2.65 ± 0.66 mV, *P*<0.01, Figure 4A). The Spearman’s correlation analysis further revealed that the mean left atrial voltage was inversely correlated with the serum LOXL2 levels (*r*^2^ = −0.49, *P*<0.01) (Figure 4B) in patients with AF.

### Univariate and multivariate analyses of predictors of AF

The univariate regression analysis model showed that serum LOXL2 [odds ratio (OR) 1.011, 95% confidence interval (CI) 1.006–1.016, *P*<0.001] and LAD (OR 1.449, 95% CI 1.222–1.717, *P*<0.001) were associated with AF. Multivariate regression analysis further demonstrated that serum LOXL2 (OR 1.013, 95% CI 1.002–1.024, *P*<0.05) and LAD (OR 1.704, 95% CI 1.131–2.568, *P*<0.01) were independent predictors of AF in this patient group (Table 2).

**Table 2:** The univariate and multivariate binomial regression analyses demonstrating the relationship between baseline characteristics and presence of AF

	Univariate analysis			Multivariate analysis		
	OR	95% CI	*P* value	OR	95% CI	*P* value
LOXL2 (pg/ml)	1.011	1.006–1.016	0.00	1.013	1.002–1.024	0.02
LAD (mm)	1.449	1.222–1.717	0.00	1.704	1.131–2.568	0.01

## Discussion

The present study reported the following results: (1) serum LOXL2 was significantly elevated in patients with AF compared with patients without AF. (2) LAD was independently correlated with serum LOXL2 levels in patients with AF. Serum LOXL2 levels were also significantly higher in AF patients with LAD ≥ 40 mm compared to those with LAD < 40 mm. (3) The level of serum LOXL2 was significantly correlated with the mean left atrial voltage in AF patients. Multivariate regression analysis further demonstrated that serum LOXL2 and LAD were independent predictors of AF. To our knowledge, the present study is the first to report the relationship between LOXL2 and the presence of AF. Since LOXL2 is an important fibrosis factor [7,10], and the pathogenesis of AF has been shown to be associated with atrial fibrosis [3–5], the present study suggests that LOXL2 levels may predict the degree of atrial fibrosis in AF patients.

The lysyl oxidase family of proteins comprises LOX and four lysyl oxidase-like proteins (LOXL1, LOXL2, LOXL3, and LOXL4). These proteins are copper- and quinone-dependent amine oxidases [11]. LOX was first described to be responsible for the covalent crosslinking of collagen and elastin in the extracellular matrix [12]. Several studies have described LOXL2 induction of tumor progression and fibrosis [13,14]. Dysregulation of LOXL2 is strongly associated with fibrotic disorders [15,16], cardiovascular diseases [17,18], and cancers [19]. One recent study focused on the relationship between LOXL2 and cardiac fibrosis in HF [8]. The researchers found that LOXL2 levels were elevated in heart tissues and serum in patients with HF, and its levels were correlated with cardiac dysfunction and HF biomarker levels. Results from human and animal studies suggest a pathogenic role of LOXL2 in cardiac fibrosis and human HF. In our study, we therefore excluded patients with factors that may affect LOXL2, such as HF, tumors, and pulmonary fibrosis, and found that LOXL2 was significantly higher in patients with AF than those without AF, indicating that LOXL2 may be involved in the pathogenesis of AF.

AF is a common cardiac rhythm disturbance and increases in prevalence with advancing age. The mechanisms causing and sustaining AF are multifactorial [2]. Left atrial remodeling, including structural, electrical, or autonomic remodeling, is known to be associated with AF [20,21]. Specifically, structural remodeling with progressive fibrosis extension within the left atrium favors the development of focal activities and reentries and initiating and maintaining AF [22]. Myocardial fibrosis is the common feature in both experimental and human AF [3]. As LOXL2 is a biomarker for fibrosis, the increased level of LOXL2 in AF patients may indicate the involvement of LOXL2 in myocardial fibrosis and the progression of AF. Numerous reports have suggested that the severity of atrial fibrosis correlates with the risk of stroke [23] and decreased response to catheter ablation [24]. Therefore, a biomarker such as LOXL2, which could reflect the degree of atrial fibrosis, may have important implications in clinical practice.

LAD is an important indicator of atrial structural remodeling in patients with AF. Previous studies have suggested that LAD size is positively correlated with atrial fibrosis [25]. Our study found that serum LOXL2 levels were significantly correlated with LAD in patients with AF and that LOXL2 levels in patients with LAD ≥ 40 mm were significantly higher than those with LAD < 40 mm, further suggesting that LOXL2 could reflect the degree of atrial fibrosis. Previous studies indicated that the atria are more sensitive to profibrotic signaling and harbor a greater number of fibroblasts than the ventricles [2]. Accordingly, our study found that LOXL2 was only correlated with atrial, but not the ventricular size in AF patients with normal heart function, suggesting that LOXL2 may be a more specific indicator for atrial fibrosis at least in AF patients with normal heart function.

A large quantity of serum inflammatory and interstitial fibrotic related markers has been previously suggested as possible biomarkers for indication the AF risk or recurrence, in particular galectin-3 [21,25–27]. However, few have documented the relationship between the LA voltage mapping data and the levels of those biomarkers. In particular, a very recent study demonstrated that galectin-3 and other biomarkers such as Pro-collagen type III N-terminal pro-peptide (PIIINP) were not related to fibrosis assessed by the LA voltage [28]. Interestingly, the electrophysiological data in our study demonstrated that the LOXL2 level was inversely correlated with the mean voltage of the left atrial in AF patients. Numerous data had convincingly demonstrated that localized atrial fibrosis can cause atrial electrograms to be of low voltage, and that fibrosis related electrophysiologic changes can be represented by electroanatomic bipolar voltage mapping [29,30]. Our data, therefore, may further provide a direct evidence between the LOXL2 and the degree of left atrial fibrosis in AF patients. In addition, persistent AF is believed to result in increased atrial fibrosis. In the present study, significant higher levels of LOXL2 were observed in Px-AF patients than those in Ps-AF patients, further demonstrating the possible higher degree of atrial fibrosis in patients with persistence AF.

In addition, the relationship between brain-type natriuretic peptide (BNP) and LOXL2 was also analyzed in our study. BNP is a neurohormone generated by cardiomyocytes in the context of numerous triggers, most notably myocardial stretch [31]. Studies have shown that BNP correlated with AF burden and was an independent predictor of recurrence after AF ablation in patients with AF [26]. However, no correlation was found between LOXL2 levels and NT-proBNP in our study. This result is supported by the concept that natriuretic peptides are markers of loading changes in response to chamber stress, while LOXL2 is a biomarker of fibrosis, which is less responsive to loading change.

There were some limitations to the study. First, the sample size is small; thus, large-scale studies are necessary to confirm our findings. Second, a follow-up study was not performed in this report. As an indicator for atrial fibrosis, it is reasonable to speculate that LOXL2 could be a candidate biomarker for guiding the recurrence of AF after cather ablation in clinic. Therefore, prospective studies should be conducted in the future to observe the effect of LOXL2 on the prognosis of patients with AF and to further explore the value of LOXL2 in assessing the success rate of AF ablation and the incidence of cardiovascular events.

In conclusion, our study demonstrated that plasma LOXL2 was significantly elevated and also strongly correlated with the degree of left atrial fibrosis in AF patients with normal left ventricular function.

## Abbreviations

AF: atrial fibrillation
ALT: alanine transaminase
BNP: brain-type natriuretic peptide
FS: fractional shortening
HF: heart failure
LAD: left atrium
LTQ: lysine tyrosylquinone
LOX: lysyl oxidase
LOX2: lysyl oxidase-like-2
LV: left ventricle
LVEDD: left ventricular end diastolic dimension
LVEF: left ventricular ejection fraction
LVESD: left ventricular end systolic dimension
NT-proBNP: N-terminal pro-B-type natriuretic peptide
PV: pulmonary vein
RAD: right atrium diameter
SCr: serum creatinine
